# Case Report: Oleandrin intoxication by inhalation in beef cattle

**DOI:** 10.3389/fvets.2025.1666317

**Published:** 2025-09-08

**Authors:** Anastasia Lisuzzo, Luca Spadotto, Michele Muraro, Sandro Mazzariol, Cinzia Centelleghe, Elena Soranzo, Silva Rubini, Carlo A. Locatelli, Mauro Dacasto, Giorgia Taio, Enrico Fiore, Matteo Gianesella

**Affiliations:** ^1^Department of Animal Medicine, Production, and Health (MAPS), University of Padua, Padua, Italy; ^2^Department of Comparative Biomedicine and Food Science (BCA), University of Padua, Padua, Italy; ^3^Veterinarian Free Practitioner, Veneto, Italy; ^4^Veterinary Service, Local Health Unit n. 6-Euganea, Padua, Italy; ^5^Istituto Zooprofilattico Sperimentale della Lombardia e dell’Emilia-Romagna, Ferrara, Italy; ^6^Toxicology Unit, Pavia Poison Control Centre-National Toxicology Information Centre, Istituti Clinici Scientifici Maugeri IRCCS, Pavia, Italy

**Keywords:** oleandrin, inhalation, intoxication, cattle, histopathology

## Abstract

Oleander (*Nerium oleander*) contains more than 30 different toxic cardiac glycosides, including oleandrin. Intoxication can occur through ingestion or inhalation in several species, including cattle. A total of 76 of 205 beef cattle died within 30 h due to the burning of oleander mowing. The burning area was located approximately 20 meters from the animal housing facility. The clinical signs before death were depression, sternal decubitus, and dyspnea. Macroscopic examination revealed cavitary effusions, diffuse edema, and hemorrhagic changes. Histopathological examination confirmed hemorrhagic and edematous findings, minimal neutrophilic infiltration and hemorrhage in the heart, centrilobular hepatic necrosis, multifocal myopathy and necrosis, and chronic bronchitis. Oleandrin was detected in the lungs, kidneys, intracardiac clot, ruminal content, and liver. The concentration of oleandrin differed among tissues and animals, supporting the hypothesis that 74 of the 76 animals died from inhalation intoxication caused by oleander. The remaining two animals, which survived the hyperacute phase, later died due to secondary oleandrin intoxication via ingestion, which aggravated their pre-existing health conditions. To the best of the authors’ knowledge, this case represents the first reported instance of inhalation intoxication by oleander in cattle.

## 1 Introduction

Oleander (*Nerium oleander*) is an ornamental plant that is widely distributed throughout the Mediterranean area. This plant contains more than 30 different toxic cardiac glycosides, including oleandrin, oleandrigenin, digitoxigenin, neriin, folinerin, and rosagenin, in each of its parts (seeds, roots, leaves, flowers, fruits, branches, and stem) (1–3). Moreover, these toxins have been reported to remain effective in fresh, dried, burned, or boiled parts of the plants (3–5). Toxic cardiac glycosides are Na^+^/K^+^-ATPase inhibitors that cause an increase in the intracellular Na^+^ concentration. This affects Na^+^/Ca^2+^ exchange channels, leading to an increase in intracellular Ca^2+^ levels. As a result, there is an increase in the resting membrane potential, with greater cell excitability and automaticity. Furthermore, an increase in extracellular K^+^ levels induces hyperkalemia, which contributes to the onset of arrhythmias and hypotension. Interference with vagal tone has also been reported, leading to slow atrioventricular conduction and ventricular arrhythmias (4, 6). As a result, cellular electrical conductivity is altered, especially at the level of the myocardium, resulting in conduction blockage, ventricular arrhythmias, and eventually complete loss of myocardial contractility or asystole (7). This condition often leads to sudden death, although the toxin dose influences the severity and evolution of clinical signs (8, 9).

Accidental oleander intoxication has been reported in several species, including pets (cat and dog), mice, rats, livestock animals (horses, cattle, sheep, goats, chickens, geese, and rabbits), donkeys, and humans (5, 10). Intoxication may result from ingestion, inhalation, or contact with mucus membranes, although ingestion is the primary route (5, 6, 11). After ingestion of the plant, toxins such as oleandrin are adsorbed and distributed to various organs, especially the liver and kidneys, where they are excreted via bile, the primary route, and urine. Moreover, oleandrin can also pass the blood–brain and blood–milk barriers (5, 6). The lethal dose has been reported to be 0.005% of body weight, or 26–45 mg/Kg, for cattle (12, 13). In addition, different species sensitivity has also been reported, with small ruminants being more resistant than cattle (6). However, establishing an exact lethal dose is complex as it depends on several factors such as the type and amount of material ingested, the concentration of toxins in it, the age and health status of the animal (3).

Inhalation of smoke produced by burning oleander has been reported to be a potential route of intoxication (3, 11). Oleandrin is a lipophilic molecule, and there is evidence that lipophilic substances can be absorbed through the lungs following nebulization (5, 14). Only a few cases of inhalation intoxication have been reported in humans (15, 16). However, to the best of the authors’ knowledge, no cases of cattle inhalation intoxication by oleander have been reported in the literature.

## 2 Case description

**Figure 1:**
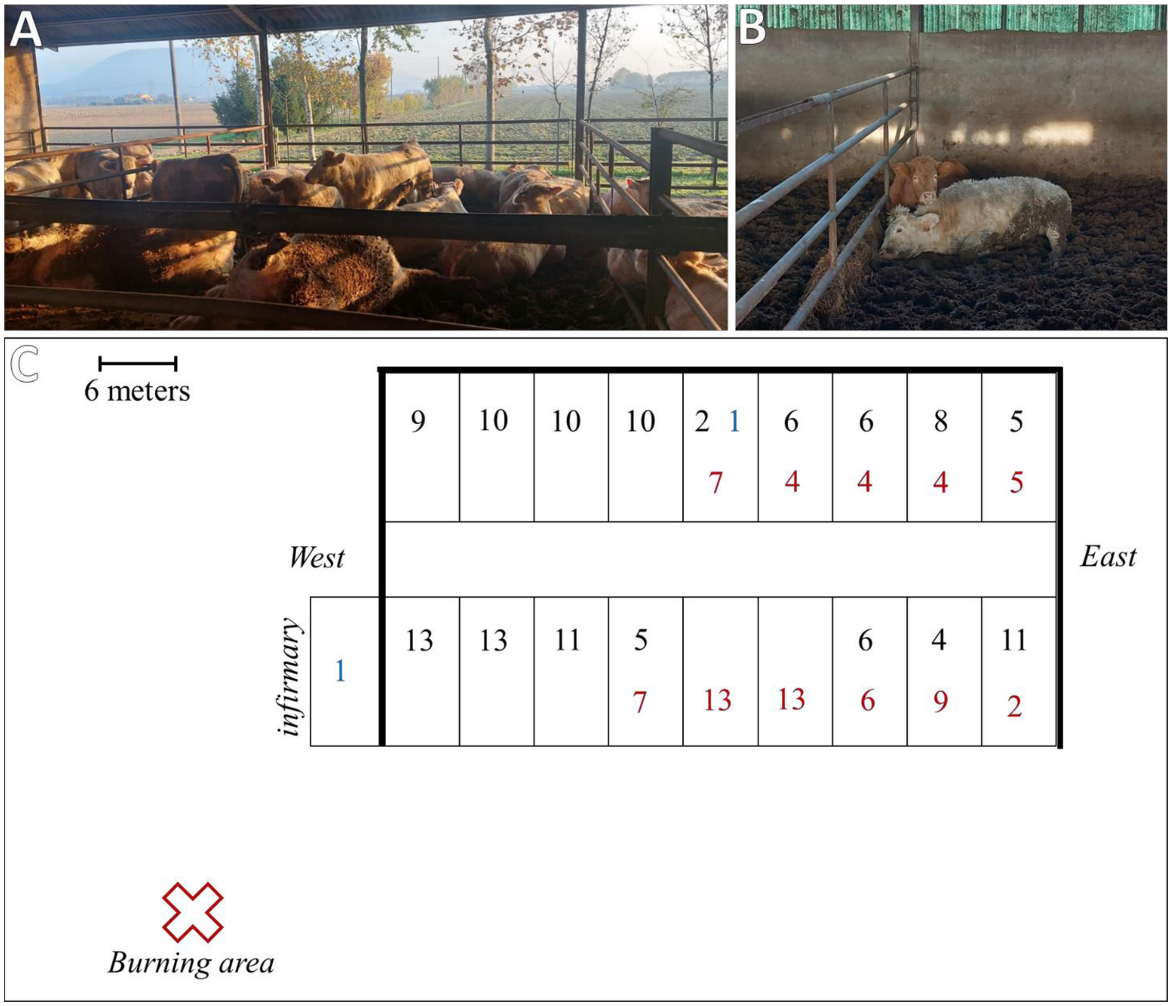
**(A)** Box conditions at the time of inspection. **(B)** One of the two clinically affected animals during inspection. **(C)** Reconstruction of the facility condition at the time of inspection: thick black lines indicate full perimeter walls; black numbers represent animals that survived without clinical signs; red numbers indicate animals that were found dead at the time of inspection; and blue numbers indicate clinical animals that died within 6 h after inspection.

The breeder of a beef cattle farm consisting of 205 animals, including Charolais breeds and Charolais × Salers crossbreeds, reported a simultaneous acute and severe reduction in sensorium, sternal decubitus, and dyspnea in approximately one-third of the animals. Consequently, the farm’s veterinarian was immediately alerted and requested support from the local veterinary service and the Department of Animal Medicine, Production, and Health (MAPS) at the University of Padua. The inspection was performed within 12 h after the onset of clinical signs. However, 74 animals were already deceased at the time of inspection, and an additional two animals with the same clinical signs died within the following 6 h (Figure 1). Moreover, the first animal that showed clinical signs after the death of the 74 animals was moved to the infirmary outside the facility.

The animals were housed in boxes containing 10–15 animals each, grouped according to age and weight. The affected animals were at different stages of the production cycle, with an age range between 10 and 18 months and an average weight of 470 Kg (min. 350–max. 600 Kg). However, both breeds and all batches of animals at different stages of the production cycle were affected. In addition, the affected boxes were located in the central and eastern parts of the facility (Figure 1). Other animals that showed no clinical signs during the hyperacute phase of the event did not develop any symptoms in the following hours/days. However, it was decided to suspend any movement from the facility as a precautionary measure.

The breeder and the farm veterinarian did not report extraordinary problems during the production cycle or clinical signs immediately preceding the event. No changes in diet or drug administration were made during the previous weeks. However, the breeder reported the burning of oleander mowing (*Nerium oleander*) within the 12 h prior to the onset of clinical signs. The burning area was located approximately 20 meters southwest of the facility. The smoke produced accumulated inside the facility, especially on the opposite side of the combustion area (the eastern part), which was characterized by the presence of the perimeter walls and closed doors. This condition corresponded with the distribution of the affected boxes. The facility doors were opened when the accumulated smoke was noticed, and the ventilation system was activated along with the removal and disposal of embers. However, feed removal and cleaning of the feeder fronts were not reported. During the inspection, a careful visual examination of the remaining feed ration did not reveal any intact or fragmented oleander leaves. Moreover, the breeder reported experiencing exhaustion and fatigue approximately 12 h after the burning of the mowing, which was temporally concurrent with the onset of the first animals’ clinical signs and required a medical visit to the hospital.

The main differential diagnoses considering the hyperacute–acute course (sudden death) in cattle (17) were as follows:

- *Lightning strike or electrocution*: However, no storm or problems in the electrical system were reported.
- *Nutritional deficiency*: Sudden death related to dietary deficiencies is typically associated with pasture-based systems, which were not used for the animals in this case report.
- *Nutritional poisoning*: Diet poisoning is related to the pasture system, or the accidental use of toxic plants in the feed. Toxins in feed components (e.g., nitrates and nitrites) were also considered. However, no dietary changes were made in the weeks before the event. Moreover, the death distribution within the facility was non-uniform. In fact, some boxes had no deceased animals (western boxes), while other boxes were completely affected. The non-homogeneous distribution suggests that the unifeed was not contaminated.
- *Ionophores or organophosphates toxicity*: No ionophore or organophosphate treatments were recorded in the previous weeks. As for nutritional poisoning, the non-normal distribution of the dead animals within the herd was poorly associated with the treatment of all animals. In addition, the affected animals varied in age and weight, which made the treatment of only one group within the herd unlikely.
- *Infectious agents*: Diseases causing septicemia or toxemia, such as anthrax, blackleg, hemorrhagic septicemia, peracute pasteurellosis, and clostridiosis, were considered. No infectious disease outbreaks were reported in and around the farm area. In addition, no new animals were introduced in the month before the event.
- *Inhalation of smoke and fumes*: This, along with severe lung edema, was considered.

Considering the anamnesis, the absence of previous clinical signs, the hyperacute–acute course, and the number of deaths, the primary differential diagnosis was oleander intoxication, followed by smoke inhalation and, lastly, infectious agents. To confirm this suspicion and exclude the other differential diagnoses, two dead animals were transported to the Veterinary Anatomical Pathology Service of the Department of Comparative Biomedicine and Nutrition (BCA) at the University of Padua for necropsy examination. The two selected animals for necropsy were evaluated based on the group they belonged to (already dead at the time of inspection or deceased shortly thereafter) and the degree of postmortem changes observed in the carcasses. Specifically, the two animals either showed no postmortem alterations or, in the case of the first group, showed only minimal alterations. However, the option to perform additional necropsies was reserved in case the findings from the initial carcasses were inconsistent. The first animal (animal A-1) died 8 h before the necropsy examination and was among the last to die, occurring approximately 30 h after the burning; while the second one (animal A-2) died 18 h before the necropsy examination and was among the first fatalities, occurring approximately 18 h after the burning.

**Figure 2:**
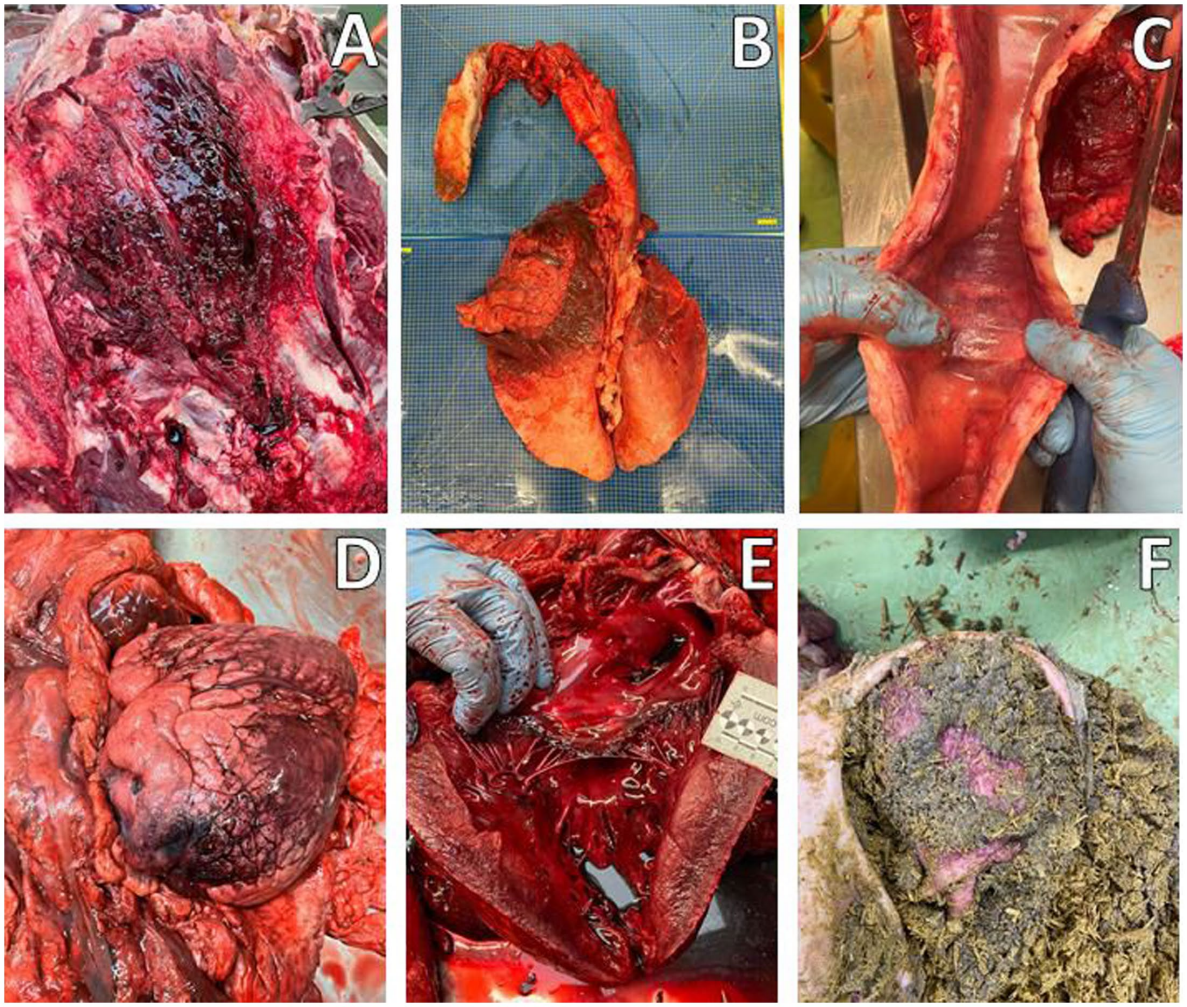
Macroscopic evaluation, Animal A-1. **(A)** Edema and hemorrhages in the subcutaneous tissue of the neck. **(B)** Congestion and hemorrhages in the cranial and middle lung lobes. **(C)** Edema and congestion in the trachea with foamy content. **(D,E)** Heart with epicardial hemorrhages. **(F)** Ruminal content.

**Figure 3:**
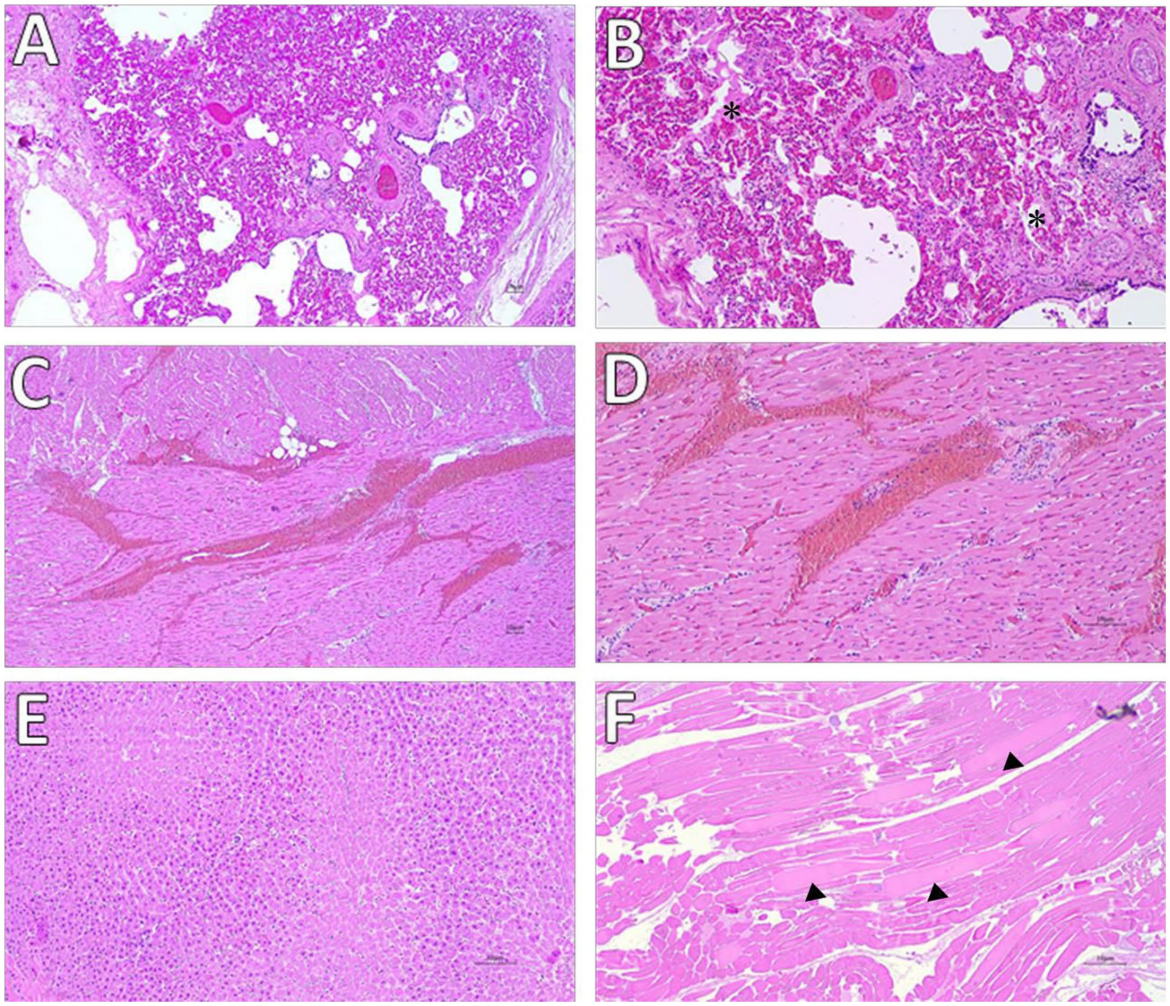
Histological evaluation of Animal A-1. **(A,B)** Lung: diffuse congestion with mild interalveolar edema and hemorrhage (asterisks), HE, 4× and 10×. **(C,D)** Heart: myocardial hemorrhages, HE, 4× and 20×. **(E)** Liver: centrilobular necrosis, HE, 20×. **(F)** Skeletal muscle: cytoplasmic degeneration, with multifocal fibers showing irregular staining and extensively eosinophilic regions alternating with more pale areas (arrowheads), HE, 4×.

Macroscopic examination revealed moderate cavitary effusions and moderate, diffuse edema (subcutaneous, peritracheal, and pulmonary) in both animals. These findings were associated with a hemorrhagic condition, characterized by petechiae and suffusions on the mucous membranes, lower airways, and muscular tissue. In addition, foamy material was observed in the trachea, but no soot was present. These findings were more pronounced in A-1, while signs of *postmortem* changes were already evident in A-2. Both subjects had no plant fragments referable to *N. oleander* in the ruminal contents (Figure 2). Therefore, during the necropsy performed on both animals according to standard procedures, tissue samples from all organs were collected for microscopic examination, including the brain, heart, trachea, lungs, liver, kidneys, and gastrointestinal tract. In addition, samples were taken for toxicological analysis, including intracardiac clot, ruminal contents, lungs, liver, and kidneys. Histopathological examination confirmed hemorrhagic and edematous findings in the investigated tissues, particularly in the heart, most notably at the epicardial level and, to a lesser extent, at the endocardial level. In case A-1, skeletal muscle damage was also observed, characterized by discoid degeneration, contraction bands, and minimal neutrophilic infiltration. Chronic bronchitis and centrilobular hepatic necrosis were also observed (Figure 3).

Toxicological analysis was carried out at the Poison Control Center and the National Center for Toxicological Information in Pavia (Italy). The analysis used the AccuDiag Digoxin competitive assay (DIG) ELISA kit, with a calibration curve constructed using oleandrin (18). Oleandrin was detected in all organs and tissues, with different distribution and concentrations, confirming the diagnosis. From a numerical point of view, A-2 exhibited higher oleandrin concentrations in the lung (774 vs. 180 ng/mL) and kidney (1,320 vs. 260 ng/mL) compared to A-1. In contrast, A-1 exhibited higher toxin concentrations in the intracardiac clot (176 vs. 55 ng/mL) and ruminal content (1,527 vs. 144 ng/mL) compared to A-2. However, similar concentrations were found in liver tissue (A-1: 186 ng/mL; A-2: 152 ng/mL).

## 3 Discussion

The clinical hyperacute–acute course observed in this case report was comparable to other clinical cases of oleander intoxication by ingestion in cattle, where clinical signs appeared between 10 and 24 h after ingestion and death occurred within 48 h (6, 8). In addition, Zorzan et al. (8) reported that some animals had a longer course of up to 10 days, probably depending on the oleander dose ingested. The clinical signs observed in this case report (depression, sternal decubitus, and dyspnea), macroscopic findings (edema and diffuse congestion, foam in trachea, and cardiac hemorrhages), and histological findings (cardiac hemorrhages, muscle tissue necrosis, alveolar edema, and hepatic necrosis) were also similar to those reported in oral oleander intoxication (6–8). However, Ceci et al. (6) and Aslani et al. (7) reported gastroenteritis with hemorrhage and esophagus inflammation, which were not found in the present clinical case. Moreover, the lung hemorrhages observed in our case were not reported in the previously mentioned studies. Clinical cases of oleander intoxication by ingestion in cattle, horses, and llamas from 1989 to 1995 in California did not report lung hemorrhages (9). In contrast, a study by Oryan et al. (19) involving experimental oleander oral poisoning in calves reported the presence of lung hemorrhages.

Oleandrin concentrations detected in deceased cattle have been reported at approximately 1–1.5 ng/mL in blood, 15 ng/mL in heart tissue, and 15–16 ng/mL in liver tissue (6, 20). A 10- to 12-fold greater concentration in liver tissues was detected in both animals evaluated in the present clinical case. Furthermore, the concentration of oleandrin in the intracardiac clot of A-1was 117 times higher than reported blood levels and 12 times higher than cardiac tissue levels in the literature; while the values were 37- and 4-fold greater, respectively, for A-2. However, this assessment must consider the different biological matrices, although the increase in oleandrin in the intracardiac clot compared to whole blood appears to be at least 30 times higher. Regarding the ruminal content, a concentration of at least 300 ng/mL was found in cattle that died from oral oleander intoxication, with leaves and fragments of the plant present in the rumen (3). The concentration detected in this clinical case was 5 times higher in animal A-1 and 2 times lower in animal A-2. To the best of the author’s knowledge, further references on oleandrin concentrations in the kidneys and lungs of cattle were not found. Tissue distribution of oleandrin in humans following oral intoxication reveals 2- to 4.5-fold and 9- to 60-fold lower concentrations in the kidneys and lungs, respectively, than those in the gastric contents. In addition, the lung toxin concentration was similar to oleandrin levels in blood or the intracardiac clot (5). In contrast, animal A-2 in this case exhibited renal and lung concentrations that were 9- and 5-fold higher, respectively, than its ruminal content, whereas animal A-1 had renal and lung concentrations 6- and 8-fold lower, respectively, than its ruminal content—consistent with findings in humans.

Considering these results, the presence of oleandrin in the ruminal content of animal A-2 (which died 18 h after the combustion) is likely unrelated to ingestion of the plant. In fact, no traces of oleander were found either in the feed residues or the ruminal contents. Furthermore, the concentration of oleandrin at that site appears to be half of what was present in another clinical case of intoxication by ingestion in cattle. In addition, the burning of the mowing and the accumulated smoke inside the facility may have caused severe stress in animals, resulting in reduced feeding behavior (21). Therefore, the presence of ruminal oleandrin in A-2 could be associated with ruminal gas contamination linked to the severe dyspnea present. Consequently, it is more likely that animal A-2 experienced oleandrin intoxication via inhalation, considering the greater lung and kidney toxin levels compared to the ruminal content. In contrast, animal A-1 (which died 30 h after the combustion) probably survived the hyperacute course and gradually resumed ingestion after the stressful event. However, some oleander glycosides are reported to be resistant to both drying and burning (3, 5). Therefore, these toxins might have contaminated the feed, resulting in oral intoxication of the two animals that survived the hyperacute course.

Inhalational oleander intoxication has been reported in humans (11, 15, 16). This clinical case was based on naturally occurring intoxication, which could not be controlled experimentally. Therefore, the exact amount of smoke generated and inhaled by the animals cannot be known, representing a limitation of the present study. However, these results support the hypothesis of oleander intoxication via inhalation and, to the best of the authors’ knowledge, represent the first reported case of such an occurrence in cattle.

## Data Availability

The raw data supporting the conclusions of this article will be made available by the authors, without undue reservation.

## References

[1] Barbosa RR Fontenele-Neto JD Soto-Blanco B. Toxicity in goats caused by oleander (*Nerium oleander*). Res Vet Sci. (2008) 85:279–81. doi: 10.1016/j.rvsc.2007.10.004, PMID: 18031775

[2] Carfora A Petrella R Borriello R Aventaggiato L Gagliano-Candela R Pietro CC. Fatal poisoning by ingestion of a self-prepared oleander leaf infusion. Forensic Sci Med Pathol. (2021) 17:120–5. doi: 10.1007/s12024-020-00338-w, PMID: 33237522 PMC7889672

[3] Rubini S Rossi SS Mestria S Odoardi S Chendi S Poli A. A probable fatal case of oleander (*Nerium oleander*) poisoning on a cattle farm: a new method of detection and quantification of the oleandrin toxin in rumen. Toxins. (2019) 11:1–9. doi: 10.3390/toxins11080442, PMID: 31349685 PMC6723884

[4] Wasfi IA Zorob O NA A k Al Awadhi AM. A fatal case of oleandrin poisoning. Forensic Sci Int. (2008) 179:e31–6. doi: 10.1016/j.forsciint.2008.05.002, PMID: 18602779

[5] Zhai J Dong X Yan F Guo H Yang J. Oleandrin: a systematic review of its natural sources, structural properties, detection methods, Pharmacokinetics and Toxicology. Front Pharmacol. (2022) 13:1–17. doi: 10.3389/fphar.2022.822726, PMID: 35273501 PMC8902680

[6] Ceci L Girolami F Capucchio MT Colombino E Nebbia C Gosetti F. Outbreak of oleander (*nerium oleander*) poisoning in dairy cattle: clinical and food safety implications. Toxins. (2020) 12:1–11. doi: 10.3390/toxins12080471, PMID: 32722138 PMC7472096

[7] Aslani MR Movassaghi AR Mohri M Abbasian A Zarehpour M. Clinical and pathological aspects of experimental oleander (*Nerium oleander*) toxicosis in sheep. Vet Res Commun. (2004) 28:609–16. doi: 10.1023/B:VERC.0000042870.30142.56, PMID: 15563108

[8] Zorzan AA Perosa FF de Cecco BS Dos Santos IR Menegatt JCO Bandinelli MB. Intoxication by *Nerium oleander* in cattle: use of immunohistochemistry for troponin C as auxiliary diagnostic method. Cienc Rural. (2024) 54:1–6. doi: 10.1590/0103-8478cr20230288

[9] Galey FD Holstege DM Plumlee KH Tor E Johnson B Anderson ML. Diagnosis of oleander poisoning in livestock. J Vet Diagnostic Investig. (1996) 8:358–64. doi: 10.1177/104063879600800314, PMID: 8844581

[10] Pugliese N Tinelli A Crescenzo G Nieddu M Baralla E Schiavone A. Poisoning by *Nerium oleander* L. in Franconia geese. Animals. (2024) 14:1–12. doi: 10.3390/ani14040612, PMID: 38396580 PMC10885877

[11] Langford SD Boor PJ. Oleander toxicity: an examination of human and animal toxic exposures. Toxicology. (1996) 109:1–13. doi: 10.1016/0300-483X(95)03296-R, PMID: 8619248

[12] Tor ER Filigenzi MS Puschner B. Determination of oleandrin in tissues and biological fluids by liquid chromatography-electrospray tandem mass spectrometry. J Agric Food Chem. (2005) 53:4322–5. doi: 10.1021/jf050201s, PMID: 15913289

[13] Soto-Blanco B Fontenele-Neto JD Silva DM Reis PFCC Nóbrega JE. Acute cattle intoxication from *Nerium oleander* pods. Trop Anim Health Prod. (2006) 38:451–4. doi: 10.1007/s11250-006-4400-x, PMID: 17243471

[14] Brillault J Tewes F. Control of the lung residence time of highly permeable molecules after nebulization: example of the fluoroquinolones. Pharmaceutics. (2020) 12:387. doi: 10.3390/pharmaceutics12040387, PMID: 32340298 PMC7238242

[15] Pillay VV Sasidharan A. Oleander and Datura poisoning: an update. Indian J Crit Care Med. (2019) 23:S250–5. doi: 10.5005/jp-journals-10071-23302, PMID: 32020998 PMC6996654

[16] Senthilkumaran S Meenakshisundaram R Michaels AD Thirumalaikolundusubramanian P. Electrocardiographic changes during inhalational oleander toxicity. J Electrocardiol. (2011) 44:470–2. doi: 10.1016/j.jelectrocard.2010.12.002, PMID: 21397908

[17] Constable PD Hinchcliff KW Done SH Grunberg W. Veterinary medicine: A textbook of the diseases of cattle, horses, sheep, pigs, and goats. St. Louis, Missouri: Elsevier (2017).

[18] Abady MM Jeong JS Kang D Jung WH Kim KY Kwon HJ. Optimization of simultaneous analysis of digoxin, desethylamiodarone, and amiodarone in serum using liquid chromatography–tandem mass spectrometry. Microchem J. (2025) 209:112724. doi: 10.1016/j.microc.2025.112724

[19] Oryan A Maham M Rezakhani A Maleki M. Morphological studies on experimental oleander poisoning in cattle. Zentralbl Veterinarmed A. (1996) 43:625–34. doi: 10.1111/j.1439-0442.1996.tb00496.x, PMID: 9011151

[20] Gosetti F Nebbia C Ceci L Carelli G Marengo E. UHPLC-MS/MS determination of oleandrin in blood and tissues of dairy cattle poisoned by oleander (*Nerium oleander*). Anal Methods. (2019) 11:5562–7. doi: 10.1039/c9ay01800j

[21] Neave HW Weary DM Von Keyserlingk MAG. Review: individual variability in feeding behaviour of domesticated ruminants. Animal. (2018) 12:S419–30. doi: 10.1017/S1751731118001325, PMID: 30109831

